# Therapeutic effect of intratumoral injections of dendritic cells for locally recurrent gastric cancer: a case report

**DOI:** 10.1186/1477-7819-12-390

**Published:** 2014-12-19

**Authors:** Masanori Kobayashi, Tomoyo Sakabe, Asako Chiba, Akihito Nakajima, Masato Okamoto, Shigetaka Shimodaira, Yoshikazu Yonemitsu, Yuta Shibamoto, Noboru Suzuki, Masaki Nagaya, The DC-vaccine study group at the Japan Society of Innovative Cell Therapy (J-SICT)

**Affiliations:** Seren Clinic Nagoya, Isokai, 4-14-2 Sakae, Naka-ku, Nagoya, 460-0008 Japan; Seren Clinic Tokyo, Isokai, 2-10-2 Shirokanedai, Minato-ku, Tokyo, 108-0071 Japan; Institute for Advanced Medical Research, Keio University School of Medicine, 35 Shinanomachi Shinjuku, Tokyo, 160-8582 Japan; Cell Processing Center, Shinshu University Hospital, 3-1-1 Asahi, Matsumoto, Nagano, 390-8621 Japan; R & D Laboratory for Innovative Biotherapeutics, Graduate School of Pharmaceutical Sciences, Kyushu University, 3-1-1 Maidashi, Higashi-ku, Fukuoka, 812-8582 Japan; Department of Radiology, Nagoya City University Graduate School of Medical Sciences, 1 Kawasumi, Mizuho-cho, Mizuho-ku, Nagoya, 467-8601 Japan; Department of Immunology, St Marianna University School of Medicine, 2-16-1 Sugao Miyamae-ku, Kawasaki, 261-8511 Japan

**Keywords:** Dendritic cell, WT1, MUC1, Immunotherapy and recurrent gastric cancer

## Abstract

An 80-year-old man with a history of gastric cancer and pulmonary emphysema underwent a distal gastrectomy for gastric cancer in 1997. In 2010, an endoscopic examination revealed a depressed-type lesion at the oral side of the anastomosis, which was diagnosed as signet-ring adenocarcinoma. Surgical management was considered, but was rejected because of obstructive and restrictive respiratory events. Chemotherapy was terminated because of adverse events. Endoscopy was used to administer intratumoral injections of dendritic cells (DCs) targeting synthesized peptides of Wilms tumor 1 (WT1) and mucin 1, cell-surface associated (MUC1). An immunohistochemical analysis of the tumor samples indicated positivity for WT1 and MUC1. One month after seven cycles of DC had been administered (between November 2010 and April 2011), no suspicious lesions were evident, and his biopsy results were normal. The patient has been in remission for 30 months. Intratumoral injections of DCs showed therapeutic effects in this patient, who could not undergo endoscopic submucosal dissection or surgery.

## Background

Gastric cancers are the second most common cause of cancer-related deaths worldwide [1]. Although surgery is the definitive treatment for gastric cancers, alternative therapeutic modalities include endoscopic submucosal dissection (ESD), chemotherapy, and radiation therapy.

Recently, it has been reported that half of all malignancies occur in patients aged over 70 years [2]. Chronic obstructive pulmonary disease (COPD), which is characterized by airflow limitation, is also common in elderly individuals [3]. In some cases, standard therapy for malignancies is not suitable because of the presence of COPD. Sakai *et al*. [4] showed that COPD was an independent risk factor for intra- and post-operative pulmonary events. In addition, Dimopoulou *et al*. [5] reported that some anti-cancer drugs induce pulmonary toxicity. For example, paclitaxel, docetaxel, and irinotecan have been reported to cause non-specific interstitial pneumonitis. In some cases, minimally invasive therapy might be required for elderly patients with COPD.

Dendritic cells (DCs) are antigen-presenting cells that are specialized for the initiation of T-cell immunity [6, 7]. DC-based immunotherapy that targets synthesized peptides has recently been used for various malignancies, including gastric cancer [8–11]. The appropriate selection of synthesized peptides is necessary to enhance the therapeutic effect of DC-based immunotherapy for gastric cancer. In 2009, the cancer antigen prioritization project of the National Cancer Institute ranked Wilms tumor 1 (WT1) and mucin 1, cell-surface associated (MUC1) as the highest and second highest priority antigens, respectively [12]. As determined by immunohistochemistry (IHC), the expressions of WT1 and MUC1 in gastric cancers were found to be 42 to 53% [13] and 93% [14], respectively.

Intratumoral administration using DC phagocytosis is a potential favorable option [15]. We used esophagogastroduodenoscopy (EGD) to administer intratumoral injections of DCs pulsed with WT1 and MUC1.

## Case presentation

**Figure 1:**
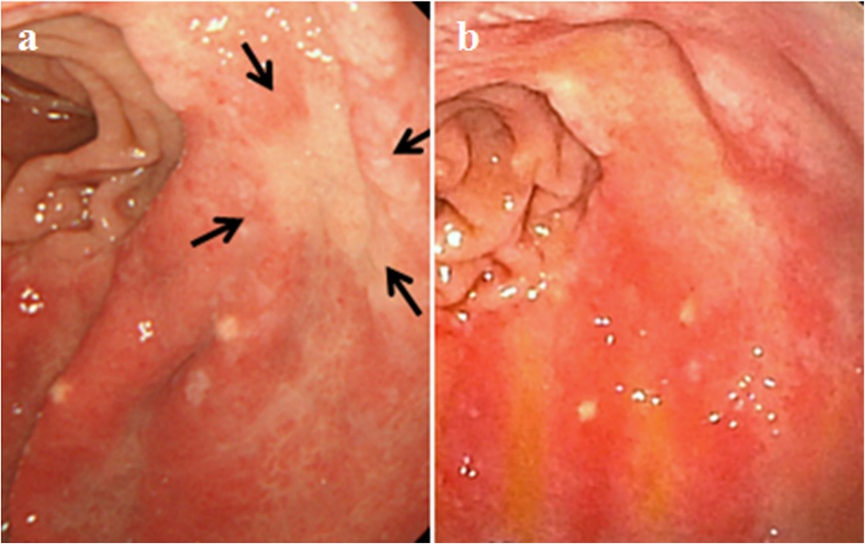
**Esophagogastroduodenoscopy (EGD) images. (a, b)** Type 0-IIc lesion (10 × 18 mm in size) on the body of the stomach near the anastomotic region (O) before vaccination **(a)**. The gastric cancer regressed to an obscure lesion one month after the final cycle of treatment **(b)**. Arrows indicate the location of the cancer.

**Figure 2:**
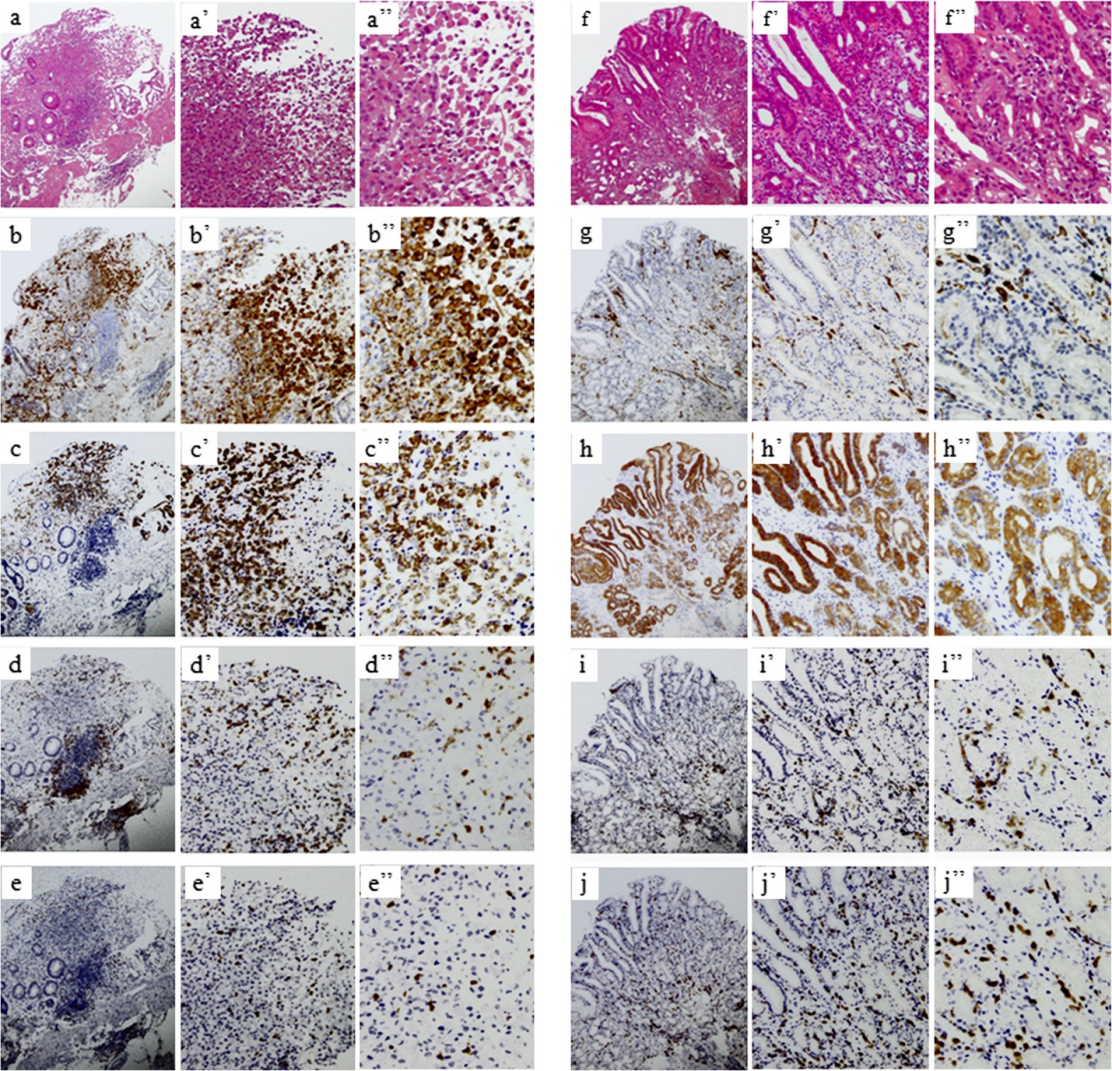
**Histological analysis of the biopsy sample.** Hematoxylin and eosin (H & E) staining **(a-a”, f-f”)** and immunohistochemistry of serial sections of tissues for Wilms tumor 1 (WT1) **(b-b”, g-g”)** and mucin 1, cell-surface associated (MUC1) **(c-c”, h-h”)**. **(a-a”, f-f”)** H & E staining before and after vaccination. H & E staining showing signet-ring cells in tissue before vaccination **(a-a”)**. One month after the final administration, pathological examination revealing normal luminal epithelial cells **(f-f”)**. **(b-b”, g-g”)** Staining for WT1 before and after vaccination. Samples positive for WT1 in approximately 60% of the cells before vaccination at the tumor site **(b-b”)** and the remarkable reduction in WT1 positivity confirmed after vaccination **(g-g”)**. **(c-c”, h-h”)** Staining for MUC1 before and after vaccination. Approximately 70% of tumor cells were positive for MUC1 before vaccination **(c-c”)** and MUC1 staining was observed in normal glands only after vaccination **(f-f”)**. Immunohistochemical staining of CD4^+^ and CD8^+^ T-cells in the biopsy samples. Serial sections of the tissues used for H & E staining prepared and stained for CD4 and CD8 **(d, e, i, j)**. **(d-d”, i-i”)** Staining for CD4 before **(d-d”)** and after vaccination **(i-i”)**. **(e-e”, j-j”)** Staining for CD8 before **(e-e”)** and after vaccination **(j-j”)**. CD4^+^ and CD8^+^ T-cells observed more frequently in normal gastric tissues after vaccination than had been observed in tumor tissues before vaccination. (Magnification: a-j 4×, a’-j’ 10×, a”-j” 20×).

An 80-year-old man with a history of gastric cancer and pulmonary emphysema underwent a Billroth I distal gastrectomy for early gastric cancer in 1997. In 2005 and 2009, he was referred for an endoscopic mucosal resection of local gastric cancer recurrence (well-differentiated tubular adenocarcinoma). In May 2010, he underwent a follow-up EGD that revealed a depressed-type lesion (10 × 18 mm in size) on the body of stomach near the anastomosis (O) (Figure 1a). A histopathological analysis of the biopsy samples revealed signet-ring adenocarcinoma (Figure 2a-a”). A computed tomography scan revealed no metastasis. Although total resection of the gastric remnant is a potentially curative therapy, this surgery was not performed in consideration of the patient’s lung dysfunction, which included obstructive and restrictive pulmonary disease (vital capacity (VC): 2.12 L; %VC: 71.9%; forced expiratory volume in 1.0 seconds: 42.9%). The patient had a 50-year history of smoking. ESD was contraindicated because the cancer was of an undifferentiated type. Therefore, treatment with TS-1 (tegafur, gimeracil, and oteracil potassium; Taiho Pharmaceutical Co, Ltd, Tokyo, Japan) was initiated in August 2010. Four weeks after TS-1 administration, treatment-related anorexia (grade 2; Common Terminology Criteria for Adverse Events, version 4.0) was observed, and hence, chemotherapy was discontinued at the request of the patient.

The methods that were used for DC preparation have been described previously [16]. The DCs were pulsed with major histocompatibility complex (MHC)-I-restricted WT1 peptide antigens, according to the patient’s human leukocyte antigen (HLA)-A pattern (HLA-A 2402; CYTWNQMNL (mutant WT1 peptide, Neo-MPS, San Diego, California, United States) and MUC1 long peptide (30 mer at 20 mg/mL; TRPAPGSTAPPAHGVTSAPDTRPAP- GSTAP; Greiner Japan, Tokyo, Japan)). The DCs were characterized using flow cytometry to ensure that they achieved the typical phenotype of mature DCs. The surface molecules that were expressed by the DCs were determined. The phenotype CD14^–/low^/HLA-DR^+^/HLA-ABC^+^/CD80^+^/CD83^+^/CD86^+^/CD40^+^/CCR7^+^ was considered to define mature DCs. The DCs were cryopreserved until the day of administration. The DC suspension was adjusted to a total volume of 1.0 mL with saline.

Six months after the gastric cancer recurrence had been diagnosed, intratumoral injections of DCs were administered using EGD between November 2010 and April 2011. This treatment was approved by the institutional review board of Isoukai (approval number: 26–1) and was conducted in accordance with the Declaration of Helsinki.

Vaccinations were repeated seven times at two to eight-week intervals (approximately 2.99 × 10^7^ cells/injection; the first four vaccinations at two-week intervals and the last three vaccinations at four- to eight-week intervals). A needle was used (diameter 25 G, length 4 mm) to administer DCs at four submucosal layer sites around the tumor. Subsequently, we administered OK432 (1-3KE) dissolved in 0.5 mL saline at two sites in the submucosal layer around the tumor. DC therapy was well tolerated and the only treatment-related adverse event was fever, with a body temperature of over 38°C for two days after the fourth vaccination. One month after the final administration, the gastric cancer was found to have regressed completely (Figure 1b). A histopathological examination of the biopsy samples revealed no signet-ring cells (Figure 2f-f”). During the 30 months following DC-based immunotherapy, no pathological recurrence has been observed. Laboratory data indicated reductions in carcinoembryonic antigen and carbohydrate antigen 19–9 levels after DC therapy, which decreased from 8.2 to 6.8 ng/mL (normal range: <5.0 ng/mL) and from 33.2 to 24.7 U/mL (normal range: <37.0 U/mL), respectively.

IHC for WT1 (Monoclonal 6 F-H2, Dako Cytomation, Carpinteria, California, United States) [13] and MUC1 (Becton Dickinson Labware; Franklin Lakes, New Jersey, United States) was performed as previously described [17], with a few modifications. IHC results were positive for WT1 in approximately 60% of signet ring cells before vaccination (Figure 2b-b”); however, the number of cells staining positive was remarkably reduced after vaccination (Figure 2g-g”). Approximately 70% of cells were positive for MUC1 before vaccination (Figure 2c-c”). After vaccination, remodeling was confirmed at the site of malignant cells, and MUC1 positivity was observed only in the normal glands (Figure 2h-h”).

No increases in the proportions of CD4^+^ and CD8^+^ T-cells were noted in the peripheral blood, based on assessments performed before and after vaccination (CD4^+^ T-cells: 43.9 to 42.5%; CD8^+^ T-cells: 29.3 to 25.5%). On the other hand, CD4^+^ and CD8^+^ T-cells were observed more frequently in the normal gastric tissue after vaccination (Figure 2i-i”, j-j”) than they had been observed in the tumor tissues before vaccination (Figure 2d-d”, e-e”).

**Figure 3:**
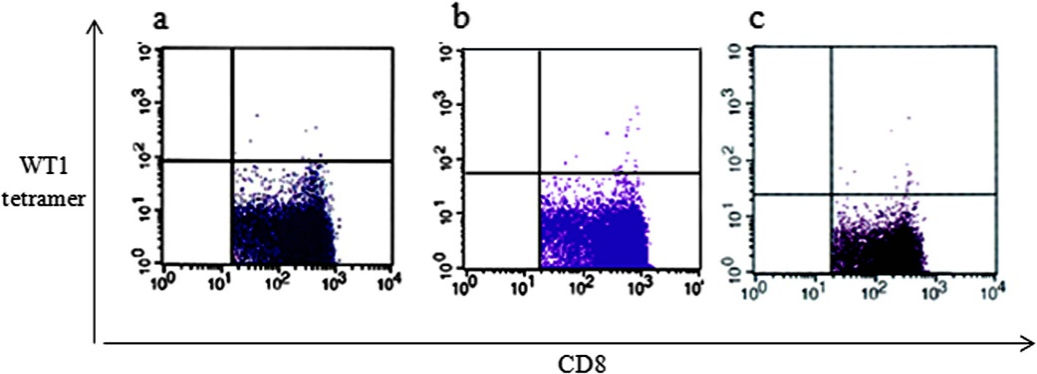
**Analysis of Wilms tumor 1 (WT1)-specific cytotoxic T lymphocytes (CTLs). (a)** Before vaccination. **(b)** After vaccination. **(c)** At 31 months after the final vaccination. WT1-specific CTL levels before vaccination increased from 0.03 **(a)** to 0.08% at the final vaccination **(b)**. At 31 months after the final vaccination, WT1-specific CTL levels increased to 0.10% **(c)**.

The frequency of WT1-specific cytotoxic T lymphocytes (CTLs) increased from 0.03 to 0.08%. The method used has been described in our previous report [16]. At 31 months after the final vaccination, the levels of WT1-specific CTLs remained high (0.10%) (Figure 3a-c).

## Discussion

In this case, restrictive pulmonary disease led the primary oncologist to opt for chemotherapy instead of surgery. However, the patient developed intolerable chemotherapy-associated anorexia, which necessitated the discontinuation of treatment. Since a few previous studies have indicated that DC immunotherapy could be safely administered to cancer patients as a minimally invasive treatment [8, 9, 18], we chose this therapy for our patient. The direct injection of DCs into tumors has been investigated in clinical studies, including those on hepatoma [19], pancreatic cancer [20], and gastric cancer [21]. Wen *et al*. [22] compared the therapeutic immunity achieved by several modes of DC injection, including intratumoral, intranodal, intravenous, and subcutaneous injections in mice. The survival rates were dramatically increased by vaccination via intratumoral injection, as compared with injections performed using other delivery methods. Pellegatta *et al*. [23] reported that, in comparison with other delivery methods, intratumoral injection of DCs may increase anti-tumor efficacy by altering the intratumoral environment and increasing T-cell-mediated immune responses. In this case, increased WT1-specific CTL levels were observed after DC immunotherapy, indicating that the DCs presented WT1 to naïve CD8^+^ T-cells in a precise manner. We suggest that gastric cancer could be a good indication for the direct injection of DCs and that the appropriate site for injection can be selected using live imaging with EGD.

Immature DCs were used for direct injections in previous studies because their phagocytic ability has been considered to be superior to that of mature DCs [24]. However, immature DCs have been shown to induce tolerance and the antigen-specific inhibition of effector T-cell function [25, 26]. Drutman and Trombetta [27] showed that mature DCs retained a robust capacity to capture soluble antigens. Furthermore, antigens internalized by mature DCs were efficiently presented on MHC class II cells and cross-presented on MHC class I cells. De Vries *et al*. [28] demonstrated that mature DCs efficiently migrate into T-cell-populated areas of the lymph nodes of melanoma patients. Taken together, these findings suggest that it might be reasonable to use mature DCs for direct injections into tumors.

IHC results for WT1 and MUC1 indicated significant changes after vaccination. Mucin is a high molecular weight glycoprotein that plays an important role in protecting the gastrointestinal tract epithelium and is normally present in abundance on the luminal surface of various secretory epithelial cells. The core peptides in the tandem repeat domain are masked in normal cells and exposed in cancer cell-associated mucins. CTLs for MUC1 only attack cancer cells with exposed tandem repeat domains [29]. In this case, MUC1-positive cells were present on the surface of the signet-ring adenocarcinoma before vaccination (Figure 2c-c”); however, MUC1-positive cells were only confirmed on normal luminal epithelial cells after DC immunotherapy, as we had expected (Figure 2h-h”).

## Conclusions

To the best of our knowledge, this is the first report on the therapeutic effects of DCs that target synthesized peptides in a patient with gastric cancer who could not undergo ESD or curative surgery.

## Consent

We provided precise explanations of the therapy to the patient and his family. They gave written informed consent for the publication of this case report and any accompanying images. A copy of the written consent is available for review by the Editor-in-Chief of this journal.

## Authors’ information

MK is a director of Seren Clinic. MN is a staff assistant at the Department of Gastrointestinal Surgery, Kanagawa Cancer Center, a director of Seren Clinic, and a professor at the Department of Immunology, St Marianna University School of Medicine. TS, AC, and AN were staff assistants at Seren Clinic. MO, SS, YY, YS, and NS are also professors at each university.

## Abbreviations

COPD: Chronic obstructive pulmonary disease
CTLs: Cytotoxic T lymphocytes
DC: Dendritic cell
EGD: Esophagogastroduodenoscopy
HLA: Human leukocyte antigen
IHC: Immunohistochemistry
MUC1: Mucin 1, cell-surface associated
WT1: Wilms tumor 1.
